# Profiling of Antimicrobial Resistance Genes and Genotype–Phenotype Associations in *Salmonella* spp. Isolated Along the Broiler Production Chain in Karnataka, India

**DOI:** 10.1155/ijfo/8152167

**Published:** 2026-04-29

**Authors:** Mohammad Nasim Sohail, Srikrishna Isloor, Doddamane Rathnamma, S. Chandra Priya, Belamaranahally M. Veeregowda, Nagendra R. Hegde, Nicola J. Williams, Csaba Varga

**Affiliations:** ^1^ Department of Pathobiology, College of Veterinary Medicine, University of Illinois Urbana-Champaign, Urbana, Illinois, USA, illinois.edu; ^2^ Department of Veterinary Microbiology, Veterinary College, KVAFSU, Bengaluru, India, kvafsu.kar.nic.in; ^3^ National Institute of Animal Biotechnology, Hyderabad, India, niab.org.in; ^4^ Department of Livestock and One Health, Institute of Infection, Veterinary and Ecological Sciences, University of Liverpool, Neston, UK, liv.ac.uk; ^5^ Carl R. Woese Institute for Genomic Biology, University of Illinois Urbana-Champaign, Urbana, Illinois, USA, illinois.edu

**Keywords:** antimicrobial resistance, antimicrobial resistance genes, broiler, colistin, ESBL, hatcheries, multidrug resistance, *Salmonella*

## Abstract

The emergence and dissemination of antimicrobial resistance genes (ARGs) in poultry‐origin *Salmonella* spp. pose a threat to food safety. This study characterized the genotypic antimicrobial resistance (AMR) in *Salmonella* spp. isolates across the broiler chicken production chain in Karnataka, India, and the co‐occurrence between AMR phenotypes and genotypes. Samples were collected from hatcheries (HAT), commercial broiler farms (CBFs), and retail meat shops (RMSs). A total of 106 *Salmonella* spp. isolates were obtained and screened for 35 ARGs belonging to seven antimicrobial classes using polymerase chain reaction (PCR). In total, 20/35 ARGs (57%) were detected in *Salmonella* spp. isolates from the entire production chain. The *tetA* (70%), *bla*_TEM_ (50%), *mcr-2–mcr-5* (29%–35%), *aac* (6 ^′^)‐Ib‐cr (21%), *qnrS* (19%), *qnrD* (18%), *cmlA1* (13%), and *sul1* (8%) were the most prevalent ARGs detected. The principal component analysis revealed distinct ARG profiles by production stage, with the highest diversity of ARGs found in RMS. All five *mcr* genes were detected in CBFs and RMSs, raising concerns about resistance to colistin, a last‐resort antibiotic for human medicine. The highest co‐occurrence was observed between doxycycline resistance and the *tetA* gene. Hierarchical clustering for AMR phenotypes and genotypes was performed using Ward′s minimum variance method. A main cluster was identified, which included co‐occurrence of resistance to tetracyclines, quinolones, aminoglycosides, and *β*‐lactams within the same isolates. Clustering of AMR phenotypes did not result in clear separation of isolates by source (HAT, CBFs, or RMSs), suggesting dissemination or persistence of shared resistance profiles across production stages. This study highlights the presence and diversity of ARGs, as well as resistance to critically important antimicrobials, and calls for interventions, including antimicrobial stewardship, enhanced biosecurity, good farming and meat processing practices, and routine surveillance to mitigate AMR.

## 1. Introduction

In recent years, the Indian poultry industry has transformed, positioning the country as a significant player in global poultry production [1]. Currently, India ranks third worldwide in egg production and fifth in chicken meat production [2]. In this growing industry, broiler chicken production plays a pivotal role, encompassing several stages: breeding, hatching, rearing, processing, and distribution [3]. The broiler production chain begins with breeding farms that supply fertilized eggs to hatcheries (HAT); these produce day‐old chicks that are then distributed to commercial broiler farms (CBFs), where chicks are raised until they reach market weight (~2 kg live body weight), after which they are processed and supplied to retail meat shops (RMSs) and processing plants [4].

Throughout this production continuum, poultry is exposed to various pathogens, among which *Salmonella* spp. remains a significant concern due to its implications for both animal and public health [5]. Some nontyphoidal *Salmonella* (NTS) serovars, such as *S.* Enteritidis and *S.* Typhimurium, can cause systemic infections in poultry, and reduced growth rates, poor feed conversion, and increased mortality, particularly in young chicks [6]. In older poultry, NTS infections often remain asymptomatic, allowing the bacteria to persist within flocks and serve as a reservoir for human infections [6]. Also, there are two poultry host‐restricted serovars, *S.* Gallinarum and *S.* Pullorum, which lead to severe diseases like fowl typhoid and pullorum disease, causing losses in affected flocks, but they are not zoonotic [7]. The majority of NTS do not produce disease in poultry; however, they pose a zoonotic risk. Several NTS serovars are an important cause of food‐borne zoonotic disease in humans worldwide, leading to approximately 95 million cases each year, resulting in an estimated 150,000 deaths, with high infection rates in low‐ and middle‐income countries, including India, due to inadequate sanitation and food safety measures [5, 8–11]. The burden of NTS is a concern in India, posing a significant health risk to local populations [12, 13]. Consumption of contaminated poultry products is a main route of NTS, leading to human bacterial gastroenteritis [14].

Antimicrobials in poultry production are administered for disease control, prevention, and growth promotion [15]. However, the misuse and overuse of antibiotics in poultry production have been considered an important driver of antimicrobial resistance (AMR) [16–18]. Antimicrobial‐resistant *Salmonella* poses a significant global health challenge, particularly due to the acquisition of antimicrobial resistance genes (ARGs) that compromise treatment efficacy [19, 20]. In recent years, an increase in the prevalence of AMR, multidrug resistance, and ARGs has been reported in NTS worldwide [8, 21, 22] as well as in India [21, 23–25], where high resistance and ARGs have been reported.

The distribution of these ARGs across different stages of the broiler production chain and its environment is particularly concerning [26]. The presence of ARGs has been studied in HAT, CBFs, and RMSs [27, 28]. However, there is limited information on studying the presence of ARGs throughout the entire broiler production chain to identify the sources of contamination and risk points for ARGs that lead to the colonization of chicks with NTS harboring resistant genes. As these birds progress through the production system, there is potential for further amplification and spread of ARGs, ultimately reaching consumers through contaminated meat products. Therefore, the objectives of this study were to assess the prevalence of ARGs in *Salmonella* spp. to important antimicrobials across the broiler production chain (HAT to CBFs and RMSs) and their environment in Karnataka, India, and identify critical control points where interventions can be most effective in reducing the AMR burden. By tracking the prevalence and distribution of ARGs from HAT to RMSs, this research aims to inform strategies to reduce the burden of AMR in poultry production and safeguard public health.

## 2. Materials and Methods

### 2.1. Location of the Study and Source of Sampling

This cross‐sectional study is a continuation of a previous study [21] that assessed phenotypic AMR across the broiler production chain and its environment in Bengaluru city and Kolar district, Karnataka, India, an important region for broiler production. The study included a total of 232 samples collected from various points along the broiler production chain The sampling framework included three broiler breeder farms (BBFs, *n* = 50), three HAT (*n* = 29), and three CBFs (*n* = 99), sampled at the beginning (Day 0), middle (Day 18), and end (Day 36) of the production cycle and three RMSs (*n* = 54). Samples were collected from three major broiler chicken integrators, companies that oversee the entire broiler production system, including BBFs, HAT, feed mills, CBFs, processing units, and marketing operations. For each integrator, a single batch was followed longitudinally through every stage of the production chain. Sampling began at the BBFs, continued at the HAT that received eggs from those breeders, then at the CBFs, which raised the resulting chicks, and finally at the RMSs, where broilers from the same batch were sold. This approach ensured that all samples represented the same flock throughout its production cycle.

At each stage, multiple sample types were collected, including water and feed, cloacal and fecal swabs, eggshell and yolk swabs, various environmental swabs (boot socks, incubators, air tunnels, and hatchers), hand swabs from hatchery personnel, carcass samples, ileal/cecal contents, and swabs from retail equipment such as cutting boards and knives. Sampling was conducted on farms without ongoing outbreaks of bacterial disease. All samples were collected using standardized aseptic sampling procedures across all production stages. All samples were transported in cold boxes and processed on the day of collection. Detailed sample types and numbers at each stage are summarized in Table 1.

**Table 1:** Samples collected from the broiler production chain.

No.	Types of samples collected	*n*
Broiler breeder farms (BBFs)		50
1	Water from the water tank of the farm (25 mL in 25‐mL 2X BPW)	6
2	Water from nipples from 30 nipples pooled into one/shed) (25 mL in 25‐mL 2X BPW)	6
3	Feed sample from feed bags (10 bags pooled into one sample/shed), around 250 g/shed	6
4	Feed sample from feeders (from 30 randomly selected areas/feeders pooled/shed) 250 g/shed	6
5	Cloacal swabs (30 birds/shed randomly selected and pooled)	9
6	Egg surface (30 randomly selected eggs/shed and pooled)	6
7	Swabs from egg yolk (30 eggs/shed pooled)	7
8	Environmental soil sample (using sterile boot socks): one pair per farm	4

Hatchery		**29**
1	Swabs from egg setting room (10 swabs pooled)	3
2	Swabs from incubator/setter (three swabs pooled)	3
3	Swabs from air tunnels and fans of incubators/setters (three swabs pooled)	3
4	Swabs from hatchers (three swabs pooled)	3
5	Swabs from hatcher′s egg tray (10 trays pooled)	3
6	Meconium swabs (10 trays pooled)	3
7	Yolk sac swab of dead chicks (10 pooled)	3
8	Hand swabs from hatchery workers (two workers)	5
9	Boot socks from the hatchery floor	3

Commercial broiler farm (CBF)		**99**
1	25 mL of water from the water tank/shed	15
2	25 mL water from 30 different nipples/shed (pooled)	25
3	25 g feed from 10 feed bags/shed (pooled)	15
4	25 g feed from 30 different feeders/shed (pooled)	25
5	Fecal swabs (30 swabs/shed pooled)	15
6	Internal environment swabs (boot socks)	9
7	External environment swabs (boot socks)	9

Retail meat shops (RMS)		**54**
1	Swabs from the surface of the cutting board (100 cm^2^)	3
2	Swabs from the cutter/knife	3
3	Meat rinsing water	3
4	Chicken carcasses (five carcasses/shop)	15
5	Ileal contents from five carcasses	15
6	Cecal contents from five carcasses	15


### 2.2. Isolates

*Salmonella* spp. was isolated as per the previously described standard methods [29]. In brief, all the samples were pre‐enriched in Buffered Peptone Water (BPW: 1.0% w/v) by incubation at 37°C for 18–24 h. After pre‐enrichment, selective enrichment was performed by transferring 1 mL of the pre‐enriched broth culture into a tube containing 9 mL of Tetrathionate Broth (TTB) and incubating at 42°C for 24–48 h. After selective enrichment, a loopful of culture was streaked onto Xylose Lysine Deoxycholate (XLD) and Brilliant Green Agar w/ Phosphates (BGA) plates. The plates were incubated at 37°C for 24 to 48 h. Four colonies per sample were selected; however, only one confirmed isolate per sample was included in the final analysis. Any duplicate isolates were excluded to avoid redundancy and ensure independence of observations. Further, each isolate was confirmed by biochemical tests using HiMedia; Hi*Salmonella* Identification Kit, KB011 and PCR targeting the *hilA* gene [30]. The isolates analyzed in this study were previously characterized phenotypically [21] and subsequently used for genotypic analysis. Confirmed isolates were preserved in nutrient broth supplemented with 30% glycerol at −80°C following phenotypic analysis for subsequent genotypic characterization.

### 2.3. Phenotypic Characterization of AMR in *Salmonella* Isolates

All confirmed *Salmonella* isolates were tested for antimicrobial susceptibility using the standard disc diffusion method recommended by the European Committee on Antimicrobial Susceptibility Testing [31]. The antibiotics used included gentamicin (10 *μ*g), amikacin (30 *μ*g), neomycin (10 *μ*g), ciprofloxacin (5 *μ*g), doxycycline (30 *μ*g), trimethoprim–sulfamethoxazole (25 *μ*g in a 1:5 ratio), chloramphenicol (30 *μ*g), ampicillin (10 *μ*g), amoxicillin–clavulanic acid (20/10 *μ*g), cefotaxime (30 *μ*g), ceftazidime (30 *μ*g), and cefpodoxime (10 *μ*g). The results were interpreted according to the EUCAST clinical breakpoints and were categorized as either susceptible or resistant to the tested antibiotics [32]. The standard broth microdilution technique was used to determine the minimum inhibitory concentration (MIC) of colistin sulfate (COL) for *Salmonella* isolates in cation‐adjusted Mueller–Hinton broth (CAMHB, HiMedia) [33].

### 2.4. Extraction of DNA From *Salmonella* Isolates

Bacterial DNA was extracted from all isolates using a DNA purification kit (QIAamp DNA Micro Kit (50) Cat. No./ID: 56304) by QIAGEN N.V., Hulsterweg 82, 5912 PL Venlo, The Netherlands. DNA concentration and purity were measured using a Nanodrop spectrophotometer, NanoDrop (Thermo Fisher Scientific Inc., 168 Third Avenue, Waltham, Massachusetts 02451, United States), with the A260/A280 and A260/A230 ratios recorded to assess purity. The extracted DNA was preserved at −20°C until further use.

### 2.5. Determination of AMR Genes in *Salmonella* Isolates

This study investigated 35 antimicrobial resistant genes (ARGs) associated with seven antimicrobial groups: aminoglycosides {(*aac (3)-IIa (aacC2) a*, *aph (3)-IIa (aphA2) a*, *aac (6* ^′^ *)-Ib*), quinolones (*qnrA*, *qnrB*, *oqxAB*, *qepA*, *qnrD*, *qnrS*, *aac (6* ^′^ *)-Ib*‐cr, *qnrC*), polymyxins (*mcr-1*, *mcr-2*, *mcr-3*, *mcr-4*, *mcr-5*), tetracyclines (*tetA*, *tetB*, *tetC*, *tetD*, *tetM*), phenicols (*catA1*, *catA2*, *cmlA1*), sulfonamides (*sul1*, *sul2*), and *β*‐lactams (*bla*_TEM_, *bla*_NDM_, *bla*_OXA_, *bla*_CTX-M_), including *bla*_CTX-M_ group‐1, *bla*_CTX-M_ group‐2, *bla*_CTX-M_ group‐19, and *bla*_CTX-M_ group‐8/25)}. Genes were selected based on a preliminary literature review, focusing on genes most commonly reported in poultry production and associated with relevant AMR patterns. Genes, primers, and PCR conditions that we investigated in our study were described previously [34–42]. Table S1 provides a detailed description of the genes, primers, and PCR conditions used for this study. PCR amplification was carried out in a 25‐*μ*L reaction mixture containing 12.5 *μ*L of Taq DNA Polymerase Master Mix RED (Ampliqon), 1 *μ*L of forward and reverse primers (10 pM), 10.5 *μ*L of nuclease‐free water, and 1 *μ*L of DNA template (~100 ng). Each PCR run included positive, negative, and no‐template controls (NTC, nuclease‐free water). PCR products were separated using 2% agarose gel electrophoresis.

### 2.6. Statistical Analysis

Statistical analysis was done using R software in R Studio (Version 2024.12.0 + 467) (Copyright 2024. The R Foundation for Statistical Computing, Posit Software, PBC) using the following packages: “aggregate,” “trend,” “tidyverse,” “ggplot,” “heatmaps. 2,” “ComplexHeatmap,” and “networkD3” [43, 44].

#### 2.6.1. Descriptive Statistics of the Prevalence of ARGs in *Salmonella* Isolates

For each sampling source (HAT, CBF, and RMS), the proportion of ARGs was calculated by dividing the number of ARG‐positive *Salmonella* isolates by the total number of isolates tested for that specific ARG. For each proportion source, exact binomial 95% confidence intervals (CIs) with the Clopper–Pearson method were calculated.

#### 2.6.2. Assessing AMR Phenotype and ARGs Coresistance and Clustering in *Salmonella* Isolates

Hierarchical clustering for AMR phenotype was performed using Ward′s minimum variance method based on Euclidean distance. The obtained dendrogram was visualized in a heat map. The second heat map, using the same method, was generated to depict the clustering of ARGs across the three sampling sources (HAT, CBFs, and RMSs) within the broiler production chain.

#### 2.6.3. Co‐Occurrence Between AMR Phenotype and ARGs in *Salmonella* Isolates

Co‐occurrence between AMR genotypes and phenotypes was examined using a Sankey plot, in which resistance genes and phenotypic resistance outcomes were represented as nodes connected by flows proportional to the number of isolates sharing each genotype–phenotype combination. The Sankey plot was used as an exploratory visualization tool to highlight patterns of concordance and co‐occurrence between resistance determinants and phenotypic antimicrobial susceptibility results.

## 3. Results

### 3.1. Prevalence of ARGs in *Salmonella* Isolates Across the Broiler Supply Chain

In this study, 106 *Salmonella* isolates were screened for the presence of 35 ARGs belonging to seven classes of antimicrobials. The result indicated that, irrespective of the sampling source and the type of ARGs, 20/35 (57%) of ARGs were present in the entire broiler chain. The proportion of ARGs was highest in RMSs (20/35, 57%), followed by CBFs (17/35, 49%), whereas only two out of 35 tested ARGs (6%) were detected in isolates from HAT. Among the nine *β*‐lactam genes studied, *bla*_TEM_ was detected in 50% of isolates, followed by the third‐generation cephalosporin resistance genes *bla*_CTX-M_ and *bla*_CTX-M_ group‐9 (4%), *bla*_SHV_ and *bla*_CTX-M_ group‐8 (2%), and *bla*_OXA_ was detected in a single isolate. None of the isolates carried the *bla*_NDM_ and *bla*_CTX-M_ group‐1 genes. Among the quinolone‐resistance genes, the highest prevalence was for *aac (6* ^′^ *)-Ib-cr*, with 21% isolates positive, followed by *qnrS* 19%, *qnrD* 18%, and *qnrB* 5%. However, none of the isolates carried *OqxAB*, *qnrA*, *qepA*, or *qnrC*. Among the five tetracycline resistance genes, only *tetA* was detected in 70% of the isolates. The phenicol resistance genes *cmlA1* and *catA2* were detected in 13% and 7% of the isolates, respectively. Among the sulfonamide resistance genes, only *sul1* was detected in 8% of the isolates. All five *mcr* genes were detected in this study, with *mcr-2* being the most prevalent (35%), followed by *mcr-3*, *mcr-4*, and *mcr*‐5 (29–31%) (Table 2).

**Table 2:** The proportion of antimicrobial resistance genes in *Salmonella* spp. isolated across the broiler production chain and crop cycle, Karnataka, India.

Antimicrobial class	Target genes	Proportion of ARGs in the broiler production chain					Proportion of ARGs in the broiler crop cycle				
		Hatcheries (*N* = 12)^a^	CBFs (*N* = 58)^a^	RMSs (*N* = 36)^a^	Total (*N* = 106)^a^	95% CI^c^	Day 0 (*N* = 28)^a^	Day 18 (*N* = 10)^a^	Day 36 (*N* = 20)^a^	Total (*N* = 58^a^	95% CI^c^
		*n*(%)^b^	*n*(%)^b^	*n*(%)^b^	*n*(%)^b^	*n*(%)^b^	*n*(%)^b^	*n*(%)^b^	*n*(%)^b^	*n*(%)^b^	
*β*‐lactams	*bla_TEM_*	0	29 (50)	24 (66.67)	53 (50)	40.13–59.87	7 (25.00)	8 (80.00)	14 (70.00)	29 (50)	36.58–63.42
	*bla*_SHV_	0	0	2 (5.56)	2 (1.89)	0.23–6.65	0	0	0	0	0–6.16
	*bla_OXA_*	0	0	1 (2.78)	1 (0.94)	0.02–5.14	0	0	1 (5.00)	1 (5.00)	0.04–.24
	*bla*_CTX-M_	0	1 (1.72)	3 (8.33)	4 (3.77)	1.04–9.38	0	0	1 (5.00)	1 (1.72)	0.04–9.24
	*bla*_CTX-M_ group‐2	0	1 (1.72)	1 (2.78)	2 (1.89)	0.23–6.65	0	0	1 (5.00)	1 (1.72)	0.04–9.24
	*bla*_CTX-M_ group‐9	0	1 (1.72)	3 (8.33)	4 (3.77)	1.04–9.38	0	0	1 (5.00)	1 (1.72)	0.04–9.24
	*bla*_CTX-M_ group‐8/25	0	0	2 (5.56)	2 (1.89)	0.23–6.65	0	0	0	0	0–6.16

Quinolone	*onrB*	0	1 (1.72)	4 (11.11)	5 (4.72)	1.55	1 (3.57)	0	0	1 (1.72)	0.04–9.24
	*onrD*	0	11 (18.97)	8 (22.22)	19 (17.92)	11.15–26.57	5 (17.86)	3 (30.00)	3 (15.00)	11 (18.97)	9.87–31.41
	*onrS*	0	7 (12.07)	13 (36.11)	20 (18.87)	11.92–27.62	0	0	7 (35.00)	7 (12.07)	4.99–23.30
	*aac(6* ^′^ *)-Ib-cr*	0	9 (15.52)	13 (36.11)	22 (20.75)	13.49–29.72	1 (3.57)	0	8 (40.00)	9 (15.52)	7.35–27.42

Polymyxins	*mcr-1*	0	1 (1.72)	1 (2.78)	2 (1.89)	0.23–6.65	1 (3.57)	0	0	1 (1.72)	0.04–9.24
	*mcr-2*	1 (8.33)	25 (43.10)	11 (30.56)	37 (34.91)	25.9–44.78	13 (46.43)	6 (60.00)	6 (30.00)	25 (43.10)	30.16–56.77
	*mcr-3*	0	25 (43.10)	8 (22.22)	33 (31.13)	22.49–40.84	12 (42.86)	6 (60.00)	7 (35.00)	25 (43.10)	30.16–56.77
	*mcr-4*	0	24 (41.38)	8 (22.22)	32 (30.19)	21.65–39.87	12 (42.86)	6 (60.00)	6 (30.00)	24 (41.38)	28.60–55.07
	*mcr-5*	0	22 (37.93)	9 (25.00)	31 (29.25)	20.81–38.87	11 (39.29)	6 (60.00)	5 (25.00)	22 (37.93)	25.51–51.63

Tetracycline	*tetA*	2 (16.67)	39 (67.24)	33 (91.67)	74 (69.81)	60.13–78.35	15 (53.57)	9 (90.00)	15 (75.00)	39 (67.24)	53.66–78.99
Phenicols	*catA2*	0	4 (6.90))	3 (8.33)	7 (6.6)	2.7–13.13	3 (10.71)	0	1 (5.00)	4 (6.90)	1.91–16.73
	*cmlA1*	0	7 (12.07)	7 (8.33)	14 (13.21)	7.41–21.17	5 (17.86)	0	2 (10.00)	7 (12.07)	4.99–23.30
Folate pathway antagonists	*sul1*	0	2 (3.45)	7 (8.33)	9 (8.49)	3.96–15.51)	0	0	2 (10.00)	2 (3.45)	0.42–11.91
Total ARGs^d^		2	17	20	20	—	12	7	15	20	—

^a^ *N* = total number of tested isolates.

^b^ *n* = total number of positive isolates.

^c^CI: exact binomial 95% confidence interval.

^d^ARGs: antimicrobial‐resistant genes.

### 3.2. Prevalence of ARGs in *Salmonella* Isolated From Different Periods of the Broiler Crop Cycle

In the broiler crop cycle, the prevalence of ARGs did not differ among the time points (Day 0, Day 18, and Day 36), except for *mcr-2*, *mcr-3*, and *mcr-4*, indicating that these genes were evenly distributed across the crop cycle. On Day 0, *qnrB*, *qnrD*, *aac (6* ^′^ *)-Ib-cr*, *mcr-1* to *mcr-5*, *tetA*, and c*atA2* were detected at prevalences ranging from 3.57% to 54%. By Day 18, in addition to these genes, the extended‐spectrum *β*‐lactamases *bla*_CTX-M_ and its groups, *catA2*, *cmlA1*, and *Sul1*, were detected at a frequency of 5%–10%. On Day 36, in addition to the genes detected on Days 0 and 18, *bla*_OXA_ and *qnrB* (2%) were identified, and the proportions of detected genes were higher on Day 36 than on Day 0 and Day 18 time points (Table 2).

### 3.3. ARGs of *Salmonella* From Different Sample Types

Among the hatchery samples, AMR genes were detected in egg trays and incubator air tunnels (*tetA*) and the yolk sacs of dead chicks (*mcr-1*). In the CBFs, AMR genes were detected in all samples except those from water tanks, including farm internal and external environments, fecal swabs, feeders, drinkers, and feed from feed bag samples. Among the RMS samples, ARGs were detected in *Salmonella* spp. isolated from five different samples: meat rinsing water, ileal and cecal contents, cutting knives, and chicken carcasses (Table 3).

**Table 3:** Antimicrobial resistance genes in *Salmonella* spp. isolated from different types of samples from the broiler production chain.

No.	Types of samples	Antimicrobial resistance genes (%)	*n*
Hatchery			29
1	Swabs from the egg setting room	0	3
2	Swabs from the incubator/setter	0	3
3	Swabs from air tunnels and fans of incubators/setters	*tetA* (33.33)	3
4	Swabs from hatchers	0	3
5	Swabs from the hatchers′ egg tray	*tetA* (33.33)	3
6	Meconium swabs	0	3
7	Yolk sac swab of dead chicks	*mcr-1* (33.33)	3
8	Hand swabs from hatchery workers	0	5
9	Boot socks from the hatchery floor	0	3

Commercial broiler farm (CBF)			99
1	Water from the water tank/shed	0	15
2	Water from 30 different water nipples/shed	*tetA* (27), *aac(6* ^′^ *)-Ib-cr* (27), *qnrS* (20), *bla*_TEM_, *mcr-2*–*5* (13)	15
3	Feed sample from 10 different feed bags/sheds	*mcr-2*–5, *cmlA2* and *cmlA1* (75), *bla*_TEM_ (44), *qnrD* and *aac(6* ^′^ *)-Ib-cr* (33), and *qnrS* (22)	15
4	Feed sample from 30 different feeders/shed	*tetA* (33), *bla*_TEM_ (27), *mcr-2*, *mcr-3*, *mcr-4* and *mcr-5* (13), and *cmlA1*(7).	15
5	Fecal swabs	*tetA* (93), *mcr-2*, *mcr-3*, *mcr-4* (60), *bla*_TEM_ (33), *qnrD* (27), *bla*_CTX-M_, *bla*_CTX-M_ group2, group9, *qnr*B, *qnrS*, *cml*A2, and *cmlA1* (7)	15
6	Internal (inside the shed) environment samples using sterile boot socks/one pair/shed	*bla*_TEM_ (87), *tetA* (53), *mcr-2* and *mcr-3* (27), *mcr-4* and *mcr-5*, *qnrD* (20), *aac(6* ^′^ *)-Ib-cr and cmlA1* (13), *qnrS* (6.67)	15
7	External (outside the shed) environment samples using sterile boot socks/farm	*mcr-2–5* (56), *tetA* and *bla*_TEM_ (44), *qnrD* and *aac(6* ^′^ *)-Ib-cr* (33), *qnrS*, *mcr-2*, *mcr-3* (22), and *mcr-1* (11)	9

Retail meat shops (RMS)			54
1	Swabs from the surface of the cutting board.	0	3
2	Swabs from the cutter/knife	*aac(6* ^′^ *)-Ib-cr*, *mcr-2*, and *tetA* (33.33)	3
3	Meat rinsing water	*tetA* (67), *qnrD*, *cmlA1*, and *sul2* (33)	3
4	Chicken carcasses (five carcasses/shop)	*bla*_TEM_ (40), *tetA*, *aac(6* ^′^ *)-Ib-cr* (26.67), *qnrS* (20), *mcr-2–5* (13.13)	15
5	Ileal contents from five carcasses	*tetA* (100%), *bla*_TEM_ (73.33), *aac(6* ^′^ *)-Ib-cr qnrS* (47), *mcr-2* (33), *mcr-3–5 qnrD* and *sul1* (27), *cml*A2 and *cmlA1* (20), *bla*_OXA_ and *qnrB* (7)	15
6	Cecal contents from five carcasses	*tetA* (60), *bla*_TEM_ (40), *mcr-2* (33), *mcr-3*–*5* (27), *qnrS* (20), *bla*_CTX-M_, *bla*_CTX-M_ group2, group9, *qnrD*, *aac(6* ^′^ *)-Ib-cr*, *cmlA1*, and *sul2* (6.67)	15


*Note:* *N* = number of samples collected.

### 3.4. Clustering of ARGs in *Salmonella* spp. Isolates

**Figure 1:**
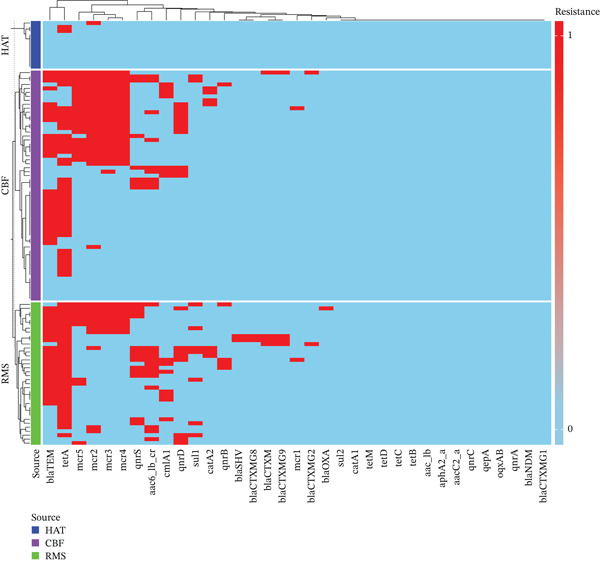
Heatmap of antimicrobial resistance gene (ARG) patterns (clusters) in *Salmonella* spp. isolated from the broiler production chain. *x*‐axis represents the ARGs: *β*‐lactamases (*bla*_TEM_, *bla*_SHV_, *bla*_OXA_, *bla*_NDM_, *bla*_CTX-M_, bla_CTX-MG1_: *bla*_CTX-M_ *group* 1, blaCTX‐MG2: *bla*_CTX-M_ *group* 2, blaCTX‐MG9: *bla*_CTX-M_ *group* 9, blaCTX‐MG8: *bla*_CTX-M_ *group* 8/25), quinolones [(*qnrA*, *qnrB*, *OqxAB*, *qepA*, *qnrD*, *qnrS*, *aac6_Ib_cr*: *aac (*6 ^′^ *)-Ib-cr*, *qnrC*)], aminoglycosides [(aacC2I_a: *(aacC*2*) a*, *aph (*3*)-IIa*, aac_ib: *(aphA*2*) a*, *aac (*6 ^′^ *)-Ib*)], polymyxins (*mcr*: *mcr*‐1, mcr2: *mcr*‐2, mcr3: *mcr*‐3, mcr4: *mcr*‐4, *mcr*5: *mcr*‐5), tetracycline (*tetA*, *tetB*, *tetC*, *tetD*, *tetM*), phenicol (*catA*1, *catA*2, *cmlA*1), and folate pathway antagonists (*sul1* and *sul2*). *y*‐axes represent *Salmonella* isolates. The red color indicates the presence of ARGs, and the blue color indicates their absence.

The clustering heat map for the distribution of ARGs in *Salmonella* spp. isolates across the broiler production chain is presented in Figure 1. The heat map was divided into three sections based on the broiler production chain: HAT, CBFs, and RMSs. Four main clusters of ARG co‐occurrence patterns were detected within the heat map columns. The first cluster (on the left side of the heat map) included beta‐lactamase and tetracycline‐resistant genes (*bla*_TEM_ and *tetA*), the most prevalent genes in the entire chain. The second cluster brought together colistin resistance genes (*mcr-2* to *mcr-5*). The third cluster (middle of the heat map) included most of the quinolones, phenicols, folate pathway antagonists, and beta‐lactams. The fourth cluster was the genes not detected in any isolates. Based on the sampling sources (HAT, CBFs, and RMSs), ARGs clustered differently. Among the hatchery isolates, only *tetA* and *mcr-2* were clustered. In contrast, the CBFs isolates had two main clusters: at the top of the heat map, a cluster of isolates harboring many ARGs with high intensity of *bla*_TEM_, *tetA*, and *mcr-2* to *mcr-5* genes, and the second cluster was mainly composed of *bla*_TEM_, *tetA* with minimum *mcr-2*, *qnrS*, and *aac (6* ^′^ *)-Ib-cr* genes. The RMS isolates had greater diversity of ARGs, with three main clusters; the first cluster at the top of the heat map was composed of *bla*_TEM_, *tetA*, and *mcr-2* to *mcr-5* genes, but the middle cluster was mostly of *bla*_TEM_, *tetA*, quinolones, and *β*‐lactam resistant genes, whereas the third cluster was of *bla*_TEM_, *tetA*, and quinolone resistance genes with fewer isolates positive for *mcr*, *sul1*, and *catA2* genes (Figure 1).

### 3.5. Distribution and Diversity of ARGs in Different Parts of the Broiler Production Chain

**Figure 2:**
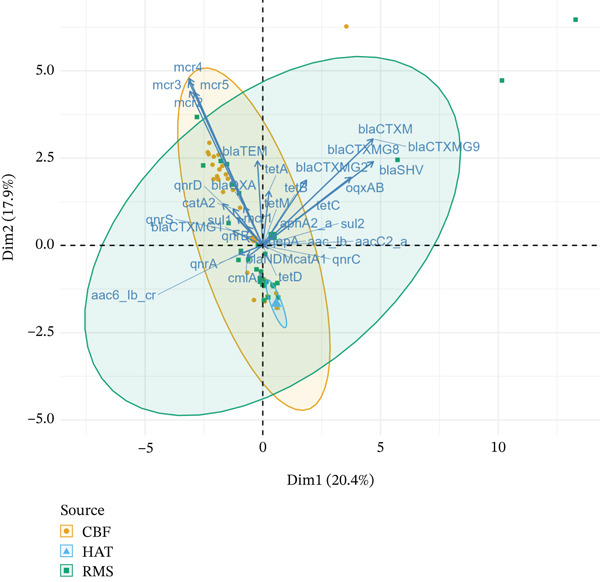
Principal component analysis (PCA) biplot of antimicrobial resistance genes (ARGs) across the broiler production chain. The PCA biplot illustrates the distribution of ARGs across isolates from hatcheries (HAT, blue triangles), commercial broiler farms (CBFs, orange circles), and retail meat shops (RMSs, green squares). Dim1 (20.4%) and Dim2 (17.9%) together explain 38.3% of the total variance in ARG presence. Vectors represent ARGs, with longer arrows indicating stronger contributions to variance. Genes such as *bla_CTXM_*, *bla_CTXMG2_*, and *oqxAB* are more associated with RMSs and CBFs samples, whereas *mcr* genes cluster with CBFs isolates. Hatchery samples are tightly grouped near the origin, reflecting lower ARG diversity. Ellipses indicate 95% confidence intervals for each source.

The principal component analysis (PCA) biplot revealed key patterns in ARG distribution across the broiler production chain (Figure 2). The first two principal components, dimensions, Dimension 1 (20.4%) and Dimension 2 (17.9%), together explained 38.3% of the total variation in ARGs presence. Hatchery samples (HAT—blue triangles) clustered tightly near the center, indicating low ARG diversity. The CBFs samples (orange circles) displayed a broader spread, suggesting higher ARG diversity, whereas the retail RMSs samples (green squares) exhibited the highest variation, indicating a significant presence of ARGs. Gene vectors highlight distinct ARG associations with each source. Tetracycline, *β*‐lactams, and quinolone resistance genes strongly contributed to Dim1, clustering toward RMS and CBF samples, suggesting that these genes were prevalent in farms and retail meat. Colistin resistance genes (*mcr-2* to *mcr-5*) were positioned toward the top‐left of Dim2, aligning more closely with CBFs isolates, which indicated a higher prevalence in CBFs. In contrast, hatchery‐associated genes (*tetA* and *mcr-2*) were near the origin, reflecting their lower overall contribution and limited presence in HAT (Figure 2).

Each point on the plot represented an isolate, and its position along these axes indicated how its ARG profile compared to others. Ellipses further illustrate the variability in the presence of ARGs. The hatchery ellipse was small, indicating minimal ARG diversity, whereas the farm and retail meat ellipses were larger, reflecting increased gene diversity at later broiler production stages (Figure 2).

### 3.6. Co‐Occurrence Between AMR Phenotypes and Genotypes

**Figure 3:**
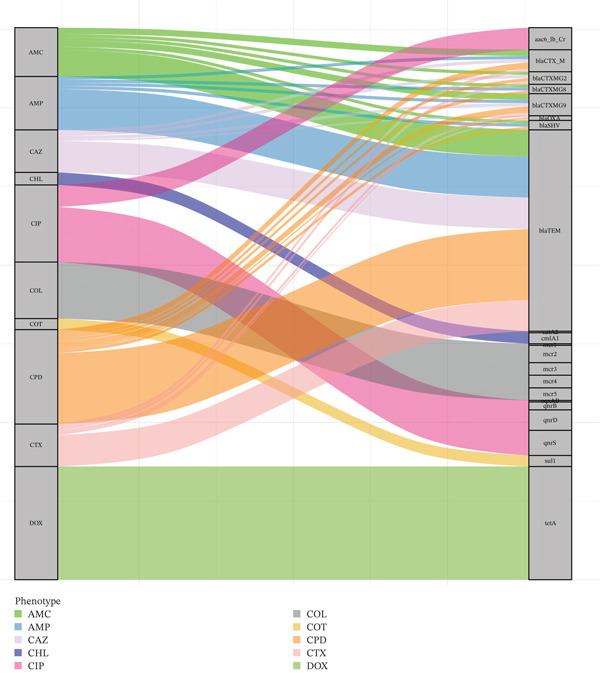
Sankey diagram visualizing the relationship between antimicrobial resistance (AMR) phenotypes and resistance genes (ARGs) in *Salmonella* spp. isolates. Phenotypic resistance is represented on the left, with corresponding ARGs on the right. The thickness of the connecting bands indicates the strength of the association, defined by the frequency of co‐occurrence. To focus on genotype–phenotype correspondence, only isolates that tested positive for both phenotypic resistance and the presence of ARGs are included.

The phenotypic AMR prevalence across the broiler production chain has been published by our research group previously [21]. This study uses the same phenotypic data and uses a Sankey diagram to visualize the co‐occurrence between AMR phenotypes and genotypes in *Salmonella* spp. isolates (Figure 3). This diagram illustrates the association of resistance characteristics by linking phenotypic resistance patterns (e.g., beta‐lactam or colistin resistance) to their corresponding ARGs. The thickness of the cords shows the strength or frequency of these associations, making it easier to identify dominant ARGs contributing to phenotypic resistance. The highest co‐occurrence was observed between doxycycline resistance and the *tetA* gene, with almost complete presence, followed by cefpodoxime resistance and the *bla*_TEM_ gene, suggesting that there may be gene variants with activity against third‐generation cephalosporins. Ciprofloxacin resistance showed the highest co‐occurrence with *qnrS*, *qnrD*, *aac (6* ^′^ *)-Ib-cr*, and *qnrB* genes. Similarly, resistance to ampicillin, amoxicillin–clavulanic acid, cefotaxime, and ceftazidime co‐occurred with *bla*_TEM_, *bla*_CTX-M_, *bla*_SHV_, and *bla*_OXA_ genes. Resistance to colistin showed a co‐occurrence with all five *mcr* genes, and resistance to chloramphenicol showed the highest co‐occurrence with *cmlA1*, followed by *catA1* genes. Resistance to trimethoprim–sulfamethoxazole co‐occurred with the *sul1* gene (Figure 3).

### 3.7. Clustering of Phenotypic AMR

**Figure 4:**
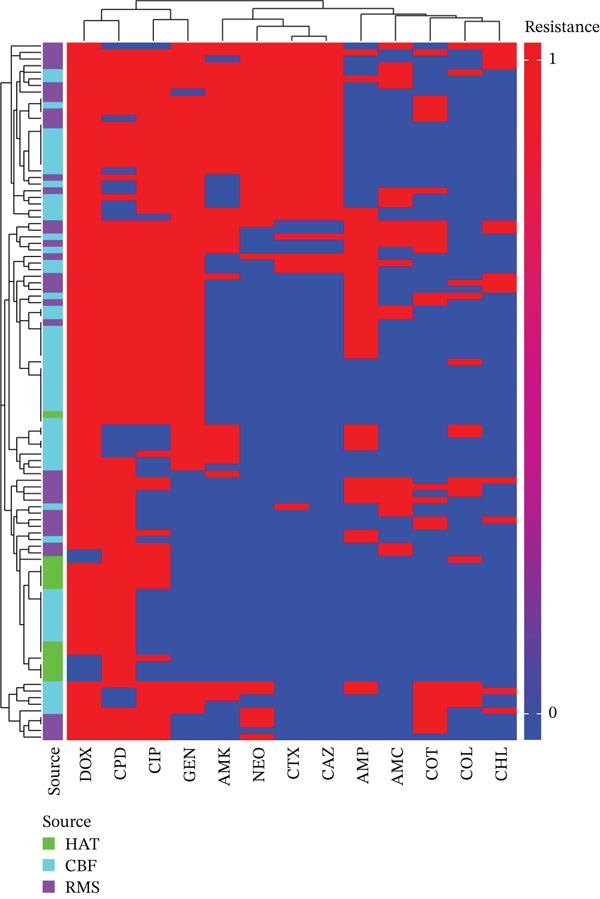
Heatmap of *Salmonella* antimicrobial resistance phenotypes in the broiler production chain. The *x*‐axis shows antimicrobials grouped by class, including aminoglycosides (gentamicin [GEN], amikacin [AMK], neomycin [NEO]), quinolones (ciprofloxacin [CIP]), tetracyclines (doxycycline [DOX]), folate pathway inhibitors (trimethoprim–sulfamethoxazole [COT]), polymyxins (colistin [COL]), phenicols (chloramphenicol [CHL]), and *β*‐lactams (ampicillin [AMP], amoxicillin–clavulanic acid [AMC], cefotaxime [CTX], ceftazidime [CAZ], cefpodoxime [CPD]). Isolates are clustered using Ward hierarchical clustering, with colors indicating resistant (red) and susceptible (blue) phenotypes.

Hierarchical clustering of the phenotypic AMR data identified three distinct clusters of co‐occurring AMR patterns (Figure 4). The first cluster consisted of resistance to doxycycline, cefpodoxime, ciprofloxacin, and gentamicin, indicating frequent co‐occurrence of resistance across tetracyclines, quinolones, aminoglycosides, and *β*‐lactams within the same isolates. A second cluster grouped resistance to the extended‐spectrum *β*‐lactams (ceftazidime and cefotaxime) with aminoglycosides (neomycin and amikacin). The third cluster comprised resistance to ampicillin and amoxicillin–clavulanic acid co‐occurring with resistance to trimethoprim–sulfamethoxazole, colistin, and chloramphenicol, representing a separate resistance profile involving *β*‐lactams, folate pathway inhibitors, polymyxins, and phenicols.

Clustering of isolates based on AMR phenotypes did not result in clear separation by source (HAT, CBF, or RMS). Isolates from HAT, CBFs, and RMSs were interspersed across multiple clusters, including those characterized by multidrug‐resistant profiles (Figure 4). Although small subclusters contained isolates predominantly from a single source, no source‐specific resistance pattern dominated the dendrogram. This distribution indicates that similar AMR phenotypes occur throughout the broiler production chain, from hatchery to retail, suggesting dissemination or persistence of shared resistance profiles across production stages rather than source‐restricted clustering.

## 4. Discussion

This study presents an evaluation of ARGs and co‐occurrences of AMR phenotypes and genotypes in *Salmonella* spp. isolates across the broiler production chain, including HAT, CBFs, and RMSs, and their environment in and around Bangalore, India. The findings provide insights into the prevalence, diversity, and distribution of ARGs. The presence of *Salmonella* spp. resistant to colistin, a last‐resort drug in human medicine, which is classified as a Highest Priority Critically Important Antibiotic (HP‐CIAs), is concerning as it poses a significant public health risk. Our study reveals a substantial presence of ARGs across the broiler supply chain, with 57% of tested samples containing ARGs. The highest diversity and frequency of ARGs were identified in RMS samples, followed by CBFs and hatchery samples. Notably, all five *mcr* genes, which encode resistance to colistin, were detected, with *mcr-2* being the most prevalent (35%), underscoring the potential role of the broiler supply chain in disseminating colistin resistance. The progressive increase in ARG prevalence from upstream (hatchery) to downstream (retail) is consistent with previous reports suggesting cumulative contamination or amplification of resistant bacteria along the production chain due to antibiotic exposure and inadequate biosecurity [14, 45, 46].

Among *β*‐lactam resistance genes, *bla_TEM_* was the most frequently detected (50%), indicating that ESBLs remain a concern in the broiler supply chain and pose a risk to the poultry industry, public health, and the environment. The detection of *bla_CTX-M_* and *bla_CTX-M_* group‐9 in a smaller proportion of isolates (4%) is consistent with the reported global emergence of CTX‐M‐type *β*‐lactamases, which have been associated with increased resistance to third‐generation cephalosporins [13, 24, 47, 48]. The presence of *bla*_SHV_ and *bla_CTX-M_* group‐8/25 in low proportions (2%) suggests limited distribution. Notably, the absence of *bla*_NDM_ and *bla*_CTX-M_ group‐1 in this study may reflect geographic variation in carbapenemase gene prevalence, and the limited presence of *bla*_CTX-M_ and *bla*_OXA_ variants, and the absence of *bla*_NDM_, indicates that carbapenemase genes are still rare in these settings, although vigilance is necessary due to their public health implications [13, 49, 50]. These findings underscore the importance of a One Health approach to managing AMR, emphasizing stringent antimicrobial stewardship, reduced use of HP‐CIAs in animal production, and enhanced surveillance programs to curb the spread of resistance genes across sectors.

Among the quinolone resistance genes, *aac (6* ^′^ *)-Ib-cr* was the most prevalent, at 20.75%, followed by *qnrS*, *qnrD*, and *qnrB*. The predominance of *aac (6* ^′^ *)-Ib-cr*, which confers resistance to aminoglycosides and fluoroquinolones, is concerning, as it can enhance resistance to clinically relevant antibiotics [51]. The presence of quinolone‐resistant *Salmonella* strains poses a public health concern [52]. The findings of this study are in agreement with a previous study in India, which identified genes such as *qnrB*, *qnrS*, and *qnrD* contributing to quinolone resistance [53]. The detection of these genes in *Salmonella* spp. strains from poultry highlights the potential for horizontal gene transfer, facilitating the spread of resistance. This may be due to their current use to treat salmonellosis and colibacillosis with fluoroquinolones, as resistance to other antimicrobials (e.g., ampicillin, trimethoprim–sulfamethoxazole, and chloramphenicol) has been increasing for several years [54]. The absence of *OqxAB*, *qnrA*, *qepA*, and *qnrC* suggests variability in the distribution of quinolone resistance determinants in the broiler supply chain and its environments.

The high prevalence of *tetA* (70%) among tetracycline resistance genes, and the increasing prevalence from 17% in HAT to 67% in CBFs and 92% in RMSs, underscores the selection pressure exerted by tetracycline use in environmental and clinical settings. There was a near‐complete co‐occurrence of the *tetA* gene with phenotypical doxycycline resistance. This finding is in line with previous studies indicating the widespread occurrence of tetracycline resistance in the commercial broiler production chain [11, 24, 48]. The phenicol resistance genes *cmlA1* (13.21%) and *catA2* (6.6%) were detected at lower frequencies, suggesting a restricted distribution of phenicol resistance mechanisms in *Salmonella* spp. isolates from the broiler supply chain.

Among sulfonamide resistance genes, only *sul1* was detected (8%), indicating a relatively low prevalence compared to other ARGs. However, the presence of *sul1* is significant, as it is often associated with mobile genetic elements, facilitating the horizontal transfer of resistance genes across bacterial populations [55].

The detection of all five *mcr* genes in this study is concerning, given their role in colistin resistance. The high prevalence of *mcr-2* (34.91%), followed by *mcr-3*, *mcr-4*, and *mcr-5* (29‐31%), suggests an increasing trend of plasmid‐mediated colistin resistance in broiler production and its environments. This finding raises concerns about the potential for *mcr-*mediated resistance in *Salmonella* and its spread to clinically relevant bacterial pathogens, further limiting treatment options for multidrug‐resistant infections. The detection of colistin resistance at each stage of the production chain is concerning, given that colistin is considered a last‐resort antibiotic in human medicine [56]. The presence of *mcr* genes in hatchery samples, even at lower frequencies, may indicate early‐life exposure or vertical transmission; however, this was not directly investigated. These findings highlight the need for stringent antimicrobial stewardship across all stages of production. Although multiplex PCR may carry a risk of nonspecific amplification or cross‐reactivity of *mcr* genes, the use of validated primers and inclusion of appropriate controls in each run minimized the likelihood of false‐positive results.

Our study findings agree with earlier studies that demonstrated higher AMR gene loads in retail meat compared to farm‐level samples, often attributed to processing contamination, cross‐contamination during transport, and suboptimal handling practices [8, 57]. Furthermore, the detection of ARGs in water, feed, and surfaces within the farms and their environment suggests multiple reservoirs and potential transmission routes, consistent with environmental dissemination of antimicrobial‐resistant strains [19, 58]. The detection of ARGs in feed, water, fecal material, and environmental samples at the CBF stage underscores the role of farm‐level practices in amplifying and disseminating AMR. Contamination of retail meat processing environments, specifically carcass rinse water and contact surfaces, further elevates the potential for consumer exposure, highlighting the risk of transmission via the food chain.

The PCA provided clear visual evidence of diverging ARG profiles: hatchery samples clustered tightly (indicating low ARG diversity), whereas CBFs and RMSs samples showed broader separation and higher gene diversity. These clusters suggest that environmental factors or management practices at later stages contribute to ARG accumulation, an observation also reported in other poultry studies [59].

The presence of clinically significant ARGs in RMSs, such as *mcr*, *bla*_CTX-M_, *bla*_SHV_, and *qnr* variants, when these were nearly absent from HAT, indicates potential contamination or antibiotic‐driven selection during rearing, slaughter, or postprocessing.

The findings of our study underscore the need for integrated surveillance and intervention strategies, especially targeting the mid and downstream points of the broiler production chain. It is critical to implement targeted control measures during the grow‐out and processing phases. Measures should include enhanced hygiene protocols, stricter biosecurity, improved feed and water quality control, biosecurity improvement, prudent antimicrobial use, and regular screening for resistance markers. Moreover, consumer‐level education and stricter poultry meat processing and handling guidelines may reduce the transmission of resistant bacteria from food to humans.

While the study provides information on ARG prevalence and distribution across the broiler production chain, some limitations must be acknowledged. The analysis was limited to a selected panel of 35 ARGs, which may not capture the full resistome present in the sampled environments. Second, the absence of whole‐genome sequencing restricted our ability to determine the genetic context and potential mobility of detected ARGs. Another limitation is the lack of serotyping data for the *Salmonella* spp. isolates, so it is unclear whether the observed AMR patterns and the presence of ARGs represent multiple serovars or a few with distinct resistance profiles. Since some serovars are more prone to AMR, this could have influenced the findings. Finally, data on actual antimicrobial use at each farm or hatchery were unavailable, which could have strengthened associations between specific practices and resistance gene prevalence. Also, the limited number of samples and their restriction to specific locations and time points may not fully capture the diversity of AMR patterns across broiler production systems, thereby limiting the generalizability of the findings. Future studies with larger sample sizes, broader geographic coverage, and longitudinal sampling across different stages of the production chain are needed. Incorporating genomic, metagenomic, and antimicrobial‐use data would further enhance understanding of AMR dynamics in poultry systems.

## 5. Conclusion

This study offers baseline data on genotypic AMR in *Salmonella* spp. across the entire broiler production and retail chain in Karnataka, India. Our findings confirm that the broiler supply chain, from HAT to RMSs, is contaminated with *Salmonella* spp. harboring several ARGs. The detection of multidrug resistance and the presence of critical ARGs, including *tetA*, *bla*TEM, *mcr*, and multiple *β*‐lactam, quinolone, sulfonamide, and chloramphenicol ARGs, highlight a public health risk. Particularly concerning is the accumulation of resistance determinants in broiler farms and retail shops, key points of consumer exposure. This might pose a threat to food safety and human health, as antimicrobial‐resistant *Salmonella* spp. and its resistance genes can be transmitted through contaminated poultry products, environmental pathways, and direct contact. Strengthened AMR surveillance, enforcement of antimicrobial use regulations, and the implementation of sustainable alternatives, such as improved biosecurity, vaccination, and good farm management practices, are needed. Educating poultry farmers and poultry supply chain stakeholders on disease prevention and antimicrobial use might reduce the emergence and transmission of antimicrobial‐resistant *Salmonella* spp.

## Author Contributions

Conceptualization and planning: M.N.S., D.R., S.I., N.R.H., and N.J.W. Funding acquisition: N.R.H., S.I., and N.J.W. Investigation: M.N.S., S.I., and S.P. Methodology: M.N.S., S.I., N.R.H., N.J.W., and C.V. Project administration: M.N.S., S.I., N.R.H., and N.J.W. Writing original draft: M.N.S. Review and editing: S.I., D.R., R.H., B.M.V., N.J.W., and C.V. Supervision: S.I., D.R., and C.V. Formal analysis and software: M.N.S. and C.V.

## Funding

This study was supported by the Indo‐UK project, Chicken or Egg: Drivers of Antimicrobial Resistance in Poultry in India, supported by the Department of Biotechnology, Ministry of Science and Technology, Government of India, BT/IN/Indo‐UK/AMR/05/NH/2018‐19, and the Economic and Social Research Council, 10.13039/501100000269, ES/S000216/1.

## Disclosure

The funders had no involvement in the design of the study, data collection, data analysis, interpretation, or the decision to submit the manuscript for publication.

## Ethics Statement

All integrators and participants in this study were anonymous, and ethical approval was granted by the Institutional Animal Ethics Committee (IAEC) of the Karnataka Veterinary, Animal, and Fisheries University (KVAFSU) (Approval Number VCH/IAEC/2019/111).

## Conflicts of Interest

The authors declare no conflicts of interest.

## Supporting information


**Supporting Information. Table S1:** Additional supporting information can be found online in the Supporting Information section. Antimicrobial resistance gene primers and PCR conditions.

## Data Availability

The original contributions presented in this study are included in the article. Further inquiries can be directed to the corresponding author.
