# Supplementary material for: Profiling of Antimicrobial Resistance Genes and Genotype–Phenotype Associations in Salmonella spp. Isolated Along the Broiler Production Chain in Karnataka, India
Source: Int J Food Sci. 2026 Apr 29;2026:8152167. doi: 10.1155/ijfo/8152167 (PMC13126426; doi:10.1155/ijfo/8152167)
Supplement: Supplementary file 1 — Supporting Information. Table S1: Additional supporting information can be found online in the Supporting Information section. Antimicrobial resistance gene primers and PCR conditions. [file IJFO-2026-8152167-s001.docx]

**Supplementary Table 1: Antimicrobial resistance genes primers and PCR conditions.**

| **Gene** | **PCR primer sequences (5′–3′)** | **Size  (bp)** | | **PCR cycling  conditions** | **References** |
| --- | --- | --- | --- | --- | --- |
| **Aminoglycoside resistance genes** | | | | | |
| *aac(3)-IIa (aacC2)a* | F: CGGAAGGCAATAACGGAG  R: TCGAACAGGTAGCACTGAG | 428 | | 94°C/5mins, 94°C/30s, 50°C/30s, 72°C/1.5 min (30 Cycles)  72°C/5mins | (Jaja et al., 2020) |
| *aph(3)-IIa (aphA2)a* | F: GAACAAGATGGATTGCACGC  R: GCTCTTCAGCAATATCACGG | 510 | |  |  |
| *aac(6’)-Ib* | F: GCGATGCTCTATGAGTGGCTA  R: CTCGAATGCCTGGCGTGTTT | 482 | | 94°C/45s, 55°C/45s  72°C/45s, (34 cycles)  72°C/5mins | (Tsukamoto et al., 2014) |
| **Quinolone resistance genes (Set I)** | | | | | |
| *qnrA* | F: CAGCAAGAGGATTTCTCACG  R: AATCCGGCAGCACTATTACTC | 631 | | 95°C/15mins,  94°C/30s  55°C/90s  72°C/90s  30 Cycles 72°C/90s | (Ciesielczuk et al., 2013) |
| *qnrB* | F: GGCTGTCAGTTCTATGATCG  R: GAGCAACGATGCCTGGTAG | 889 | |  |  |
| *OqxAB* | F: CCGCACCGATAAATTAGTCC  R: GGCGAGGTTTTGATAGTGGA | 313 | |  |  |
| *qepA* | F: GCAGGTCCAGCAGCGGGTAG  R: CTTCCTGCCCGAGTATCGTG | 218 | |  |  |
| **Quinolone resistance genes (Set I)** | | | |  |  |
| *qnrD* | F: CGAGATCAATTTACGGGGAATA  R: AACAAGCTGAAGCGCCTG | 582 | |  |  |
| *qnrS* | F: GCAAGTTCATTGAACAGGGT  R: TCTAAACCGTCGAGTTCGGCG | 427 | |  |  |
| *aac(6’)-Ib-cr* | F: TTGGAAGCGGGGACGGAC  R: ACACGGCTGGACCATA | 260 | |  |  |
| *qnrC* | F: GCAGAATTCAGGGGTGTGAT  R: AACTGCTCCAAAAGCTGCTC | 118 | |  |  |
| **Phenicol resistance genes** | | | | | |
| *catA1* | F: AGTTGCTCAATGTACCTATAACC  R: TTGTAATTCATTAAGCATTCTGCC | 320 | | 94°C/5mins  94°C/30s  50°C/30s  72°C/1.5 min  For 30 Cycles 72°C/5mins | (Jaja et al., 2020) |
| *catA2* | F: ACACTTTGCCCTTTATCGTC  R: TGAAAGCCATCACATACTGC | 543 | |  |  |
| *cmlA1* | F: CACCAATCATGACCAAG  R: GGCATCACTCGGCATGGACATG | 115 | |  |  |
| **The *mcr* genes set I** | | | | | |
| *mcr-1* | F: ATGCCAGTTTCTTTCGCGTG  R: TCGGCAAATTGCGCTTTTGGC | 502 | 94°C/4mins  94°C/5sec  59°C/20s  For 30 Cycles 72°C/5mins | | (Lescat et al., 2018) |
| *Mcr-5* | F: GGACGCGACTCCCTAACTTC  R: ACAACCAGTACGAGAGCACG | 608 |  |  |  |
| **The *mcr* genes set II** | | |  |  |  |
| *mcr -2* | F: GATGGCGGTCTATCCTGTAT  R: AAGGCTGACACCCCATGTCAT | 379 |  |  |  |
| *mcr-3* | F: ACCAGTAAATCTGGTGGCGT  R: AGGACAACCTCGTCATAGCA | 296 |  |  |  |
| *mcr-4* | F: TTGCAGACGCCCATGGAATA  R: GCCGCATGAGCTAGTATCGT | 207 |  |  |  |
| **Tetracycline resistance genes** | | | | | |
| *tetA* | F: GCTACATCCTGCTTGCCTTC  R: CATAGATCGCCGTGAAGAGG | 201 | 94°C/5mins  94°C/1min  55°C/1min  72°C/1.5 min  For 35 Cycles 72°C/5mins | | (Jaja et al., 2020) |
| *tetB* | F: TTGGTTAGGGGCAAGTTTTG  R: GTAATGGGCCAATAACACCG | 359 |  |  |  |
| *tetC* | F: CTTGAGAGCCTTCAACCCAG  R: ATGGTCGTCATCTACCTGCC | 418 |  |  |  |
| *tetD* | F: AAACCATTACGGCATTCTGC  R: GACCGGATACACCATCCATC | 300 |  |  |  |
| *tetM* | F: AGT GGA GCG ATT ACA GAA  R: CAT ATG TCC TGG CGT GTC TA | 158 |  |  |  |
| **Sulphonamide resistance genes** | | | | | |
| *sul1* | F: TTCGGCATTCTGAATCTCAC  R: ATGATCTAACCCTCGGTCTC | 822 | 94°C/5mins, 94°C/1min  55°C/1mins, 72°C/1.5min  (35 Cycles), 72°C/5mins | | (Jaja et al., 2020) |
| *sul2* | F: CGGCATCGTCAACATAACC  R: GTGTGCGGATGAAGTCAG | 625 |  |  |  |
| ***β* Lactams resistance genes set I** | | | | | |
| *bla*_TEM_ | F: CATTTCCGTGTCGCCCTTATTC  R: CGTTCATCCATAGTTGCCTGAC | 800 | 95°C/12mins  95°C/30s  55°C/30s 72°C/1min  For 30 Cycles  72°C/5mins | | (Dallenne et al., 2010) |
| *bla*_SHV_ | F: AGCCGCTTGAGCAAATTAAAC  R: ATCCCGCAGATAAATCACCAC | 713 |  |  |  |
| *bla*_OXA_ | F: GGCACCAGATTCAACTTTCAAG  R: GACCCCAAGTTTCCTGTAAGTG | 564 |  |  |  |
| *bla*_NDM_ | F: TGGATCAAGCAGGAGATCAA  R: GGCCGGGGTAAAATACCTT | 250 |  |  | (Buelow et al., 2018) |
| *bla*_CTX-M_ | F: ATGTGCAGCACCAGTAAAGTGATGGC  R: TGGGTAAAGTAAGTCACCAGAATCAGCGG | 593 |  |  | (Boyd et al., 2004; Nakhaei Moghaddam et al., 2014) |
| ***β* Lactams resistance genes set II** | | |  |  |  |
| *bla*_CTX-M_ group 1 | F: TTAGGAAGTGTGCCGCTGTA  R: CGATATCGTTGGTGGTGCCAT | 688 |  |  | (Dallenne et al., 2010) |
| *bla*_CTX-M_ group 2 | F: CGTTAACGGCACGATGAC  R: RCGATATCGTTGGTGGTGCCAT | 404 |  |  |  |
| *bla*_CTX-M_ group 9 | F: TCAAGCCTGCCGATCTGGT  R: TGATTCTCGCCGCTGAAG | 561 |  |  |  |
| *bla*_CTX-M_ group 8/25 | F: AACACGCAGACGCTCTAC  R: TCGAGCCGGAACGTGTCAT | 326 |  |  |  |
